# Frequency-specific cortical subnetworks support fast human swallowing

**DOI:** 10.1038/s42003-026-10751-6

**Published:** 2026-08-07

**Authors:** Paul Muhle, Jonas von Itter, Anne Jung, Bendix Labeit, Ivy Cheng, Inga Claus, Andreas Wollbrink, Joachim Gross, Rainer Dziewas, Sonja Suntrup-Krueger

**Affiliations:** 1 Department of Neurology, University Hospital Münster, Münster, Germany; 2 Institute for Biomagnetism and Biosignalanalysis, University Hospital Münster, Münster, Germany; 3 Department of Neurology, Heinrich Heine University Düsseldorf, Düsseldorf, Germany; 4 Academic Unit of Human Communication, Learning, and Development, The University Of Hong Kong, Hong Kong, Hong Kong; 5 Centre for Gastrointestinal Sciences, Faculty of Biology, Medicine and Health, University of Manchester, Manchester, UK; 6 Department of Neurology, Klinikum Osnabrück, Osnabrück, Germany

**Keywords:** Motor cortex, Sensory processing, Neural circuits

## Abstract

Fast, highly constrained sensorimotor acts require rapid coordination of distributed cortical systems on subsecond timescales. Here, we used source-resolved magnetoencephalography to characterise time-locked cortical connectivity during voluntary swallowing in 74 healthy adults. Cluster-based network statistics revealed focal swallowing-related connectivity changes confined to anatomically selective subnetworks. Undirected phase-lagged connectivity identified theta- and low-gamma weighted phase lag index (wPLI) effects involving somatosensory, motor, supramarginal, and insular regions. Directed connectivity revealed sparse high-gamma phase slope index (PSI) subnetworks centred on the primary somatosensory cortex and anterior insula. Time-window analyses demonstrated temporally evolving low-gamma interactions between posterior parietal and insular regions, while laterality analyses showed rightward theta-band directed asymmetries during later swallowing phases. In contrast, global graph-theoretical metrics and node-level hub measures remained largely stable after correction for multiple comparisons. These findings indicate that voluntary swallowing is supported by focal, frequency-specific, and temporally structured cortical interactions rather than broad global network reconfiguration.

## Abstract (web summary)

Source-resolved magnetoencephalography reveals frequency-specific cortical subnetworks that coordinate rapid human swallowing and provides a systems-level framework for cortical sensorimotor control.

## Introduction

Complex behaviour emerges from the rapid integration of sensory and motor processes across distributed cortical networks rather than from isolated regional activations. While network neuroscience has provided important insights into large-scale brain organisation during sustained cognitive and motor states^1,2^, considerably less is known about how transient cortical interaction patterns are expressed during fast sensorimotor behaviours unfolding on subsecond timescales. Such behaviours require continuous sensorimotor integration, precise motor execution, and rapid transitions between interaction patterns^1,3–5^, yet their event-level cortical network organisation in humans remains incompletely understood.

Characterising these rapid network dynamics requires a behavioural model that is (i) fast and stereotyped, (ii) precisely time-lockable to physiological markers, and (iii) under continuous sensorimotor control. Many commonly studied motor behaviours either unfold over longer timescales, lack reliable physiological markers for event locking, or involve substantial variability across executions^1,4,6^. Voluntary human swallowing fulfills these criteria particularly well. It is a fast, highly stereotyped behaviour that depends on continuous sensory feedback and precise motor coordination^7^, while remaining reliably time-locked using peripheral electromyographic markers^8,9^. This combination enables the resolution of short-lived, event-locked cortical network interactions that are difficult to capture during more extended or variable forms of behaviour in humans^7,10–12^.

To capture such rapidly evolving network dynamics, methodological approaches must resolve interactions across time, frequency, and directionality at the scale of individual sensorimotor acts. Magnetoencephalography (MEG) provides millisecond temporal resolution required to track subsecond changes in cortical communication, while source-resolved analysis improves spatial specificity and enables the investigation of interactions between distributed cortical regions^13,14^. Frequency-resolved connectivity measures enable the dissociation of distinct modes of large-scale coordination, whereas directed metrics provide insight into the temporal ordering of inter-regional interactions beyond undirected coupling^15–17^. When combined with graph-theoretical analysis, these approaches permit a multilevel characterisation of transient interaction patterns, encompassing both pairwise connectivity changes and tests of whether the changes extend to global topological reconfiguration^18,19^. Together, this framework enables a principled, multiscale investigation of transient cortical interactions that emerge at the level of individual sensorimotor acts.

**Fig. 1:**
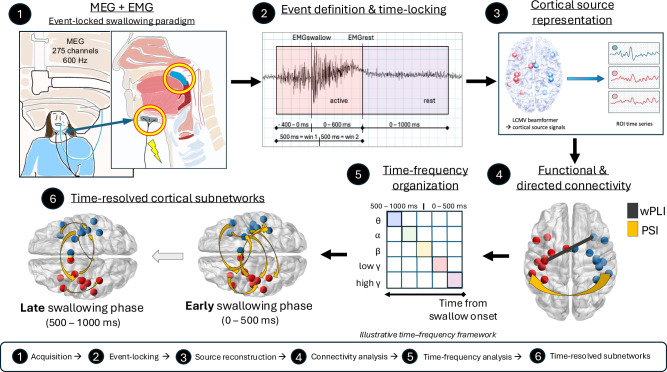
Event-locked MEG connectivity framework for resolving transient cortical swallowing subnetworks. Overview of the experimental and analytical pipeline used to investigate transient cortical network organisation during voluntary human swallowing. **(1)** Whole-head MEG (275 channels, 600 Hz) and submental EMG were recorded during self-paced swallowing. **(2)** Swallowing-related (EMGswallow/EMG2) and post-swallow control (EMGrest/EMG3) intervals were defined relative to pharyngeal swallow onset derived from the EMG signal. **(3)** Source reconstruction was performed using an LCMV beamformer to obtain cortical ROI time series. **(4)** Functional and directed connectivity were estimated using debiased weighted phase lag index (wPLI) and phase slope index (PSI). **(5)** Connectivity was analysed across canonical frequency bands and temporally resolved swallowing windows. **(6)** Time-resolved cortical subnetworks were visualised for early (0–500 ms) and late (500–1000 ms) swallowing phases. The figure illustrates the conceptual workflow of the event-locked, time–frequency–resolved source-connectivity framework used throughout the study. Schematic brain renderings and subnetworks are illustrative and not intended to depict exact statistical results.

Here, we focus on the network-level organisation of fast sensorimotor behaviour at the level of individual events, using an explicitly event-locked, time–frequency–resolved analytical framework (Fig. 1), instantiated here by voluntary human swallowing. This approach enables the characterisation of transient and frequency-specific cortical interaction patterns associated with individual swallowing events.

## Results

### Frequency-specific sensorimotor subnetworks emerged during swallowing

**Fig. 2:**
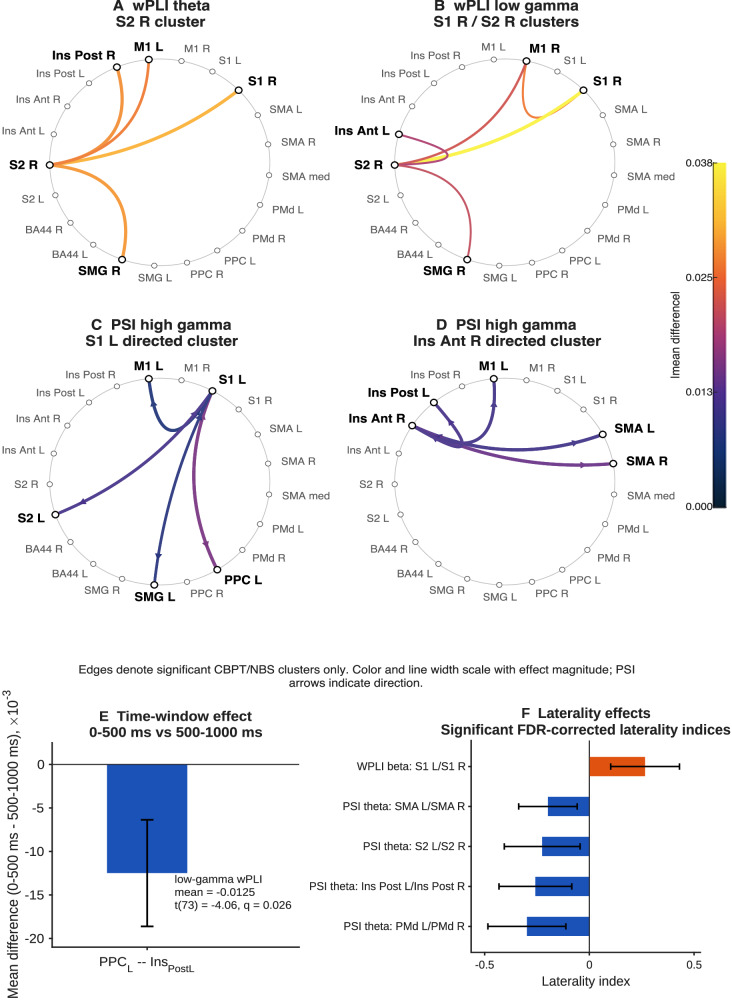
Cluster-corrected and time-resolved swallowing-related connectivity effects. **A**,**B** Significant swallowing-related weighted phase lag index (wPLI) subnetworks identified using cluster-based permutation testing (CBPT; 5000 permutations, cluster-corrected *p* < 0.05). Displayed subnetworks represent seed-to-ROI clusters showing significant within-subject differences between swallowing (EMGswallow) and control (EMGrest) conditions. Edge thickness reflects relative effect magnitude within the significant cluster. **C**,**D** Significant phase slope index (PSI) seed-to-ROI subnetworks showing statistically significant directed interaction effects during swallowing. Arrows indicate estimated directionality of phase-based interactions. PSI reflects statistical directionality estimates rather than direct causal interactions. **E** Time-resolved low-gamma wPLI difference between left posterior parietal cortex (PPC_L) and left posterior insula across early and late swallowing phases. Error bars represent standard error of the mean. Statistical significance was assessed using paired-sample t-tests with FDR correction. **F** Significant laterality indices (LI) during swallowing. Positive values indicate relative leftward predominance, whereas negative values indicate relative rightward predominance of connectivity estimates. For wPLI, positive LI values indicate relatively stronger left-hemispheric coupling; for PSI, negative values indicate relatively rightward directed phase-lag asymmetry. Full statistical results are reported in Supplementary Data 1–4. Sample size: *n* = 74 participants.

Cluster-based network statistics revealed frequency-specific alterations of cortical connectivity during swallowing, predominantly involving seed-to-ROI subnetworks centred on sensorimotor and insular regions. Whole-network ROI×ROI analyses did not reveal additional cluster-corrected effects. For weighted Phase Lag Index (wPLI), significant clusters were identified in the theta and low-gamma bands. In the theta band, a right secondary somatosensory cortex seed (S2_R) showed a significant connectivity cluster involving the left primary motor cortex (M1_L), the right primary somatosensory cortex (S1_R), the right supramarginal gyrus (SMG_R), and the right posterior insula (cluster *p* = 0.0096; Fig. 2A). All edges within this cluster showed lower wPLI during swallowing compared with the control condition, consistent with altered phase-lagged coupling within this sensorimotor–insular subnetwork.

In the low-gamma band, two significant wPLI clusters were observed. A cluster seeded in S1_R involved M1_R and S2_R (cluster p = 0.0498), while a second cluster seeded in S2_R involved M1_R, S1_R, SMG_R, and left anterior insula (cluster *p* = 0.0062; Fig. 2B). The strongest edge-level effect within these subnetworks was observed for the S1_R – S2_R connection.

Directed connectivity assessed by Phase Slope Index (PSI) revealed significant high-gamma seed-to-ROI clusters. A left S1 seed showed directed interactions with M1_L, the left posterior parietal cortex (PPC_L), SMG_L, and S2_L (cluster *p* = 0.0204; Fig. 2C). A second high-gamma PSI cluster was centred on the right anterior insula and included M1_L, the left and right supplementary motor areas (SMA_L, SMA_R), and left posterior insula (cluster *p* = 0.0392; Fig. 2D). Together, these findings indicate anatomically selective and frequency-specific interaction structure of cortical swallowing networks involving sensorimotor, parietal, and insular regions. Full cluster-level and edge-level statistics are provided in Supplementary Data 1 and 2.

### Time-resolved analyses revealed temporally structured connectivity dynamics

To assess whether connectivity patterns evolved across the swallowing sequence, we compared early and late time windows within the swallowing epoch. Comparison of the early and late windows identified one significant low-gamma wPLI effect between PPC_L and the left posterior insula. Connectivity was lower in the early than in the late window (mean difference = −0.0125, t(73) = − 4.06, *q* = 0.0255; Fig. 2E). No additional FDR-significant effects were observed in the 250-ms window analysis. Exploratory edgewise FDR-corrected effects are summarised in Supplementary Data 3. These findings indicate temporally evolving interactions between posterior parietal and insular regions across the swallowing sequence.

### Laterality analyses identified rightward theta-band directed asymmetries

Laterality analyses revealed significant hemispheric asymmetries during swallowing. In the beta band, wPLI showed a significant laterality index for S1_L/S1_R in the late quarter window (750–1000 ms), indicating lateralized somatosensory coupling (LI = 0.266, t(73) = 3.23, *q* = 0.0187; Fig. 2F).

For PSI, significant theta-band laterality effects were found in the late (500–1000 ms) window for SMA, the dorsal premotor cortex (PMd), S2, and posterior insula. These effects showed negative laterality indices, indicating a relative rightward predominance of directed interactions for these regions: SMA (LI = −0.198, *q* = 0.0203), PMd (LI = −0.298, *q* = 0.0203), S2 (LI = −0.225, *q* = 0.0388), and posterior insula (LI = −0.258, *q* = 0.0203). Together, these findings indicate temporally structured hemispheric asymmetries of directed cortical interactions during later stages of swallowing. Full laterality statistics are reported in Supplementary Data 4.

### Exploratory whole-network visualisations illustrated distributed cortical interaction structure

**Fig. 3:**
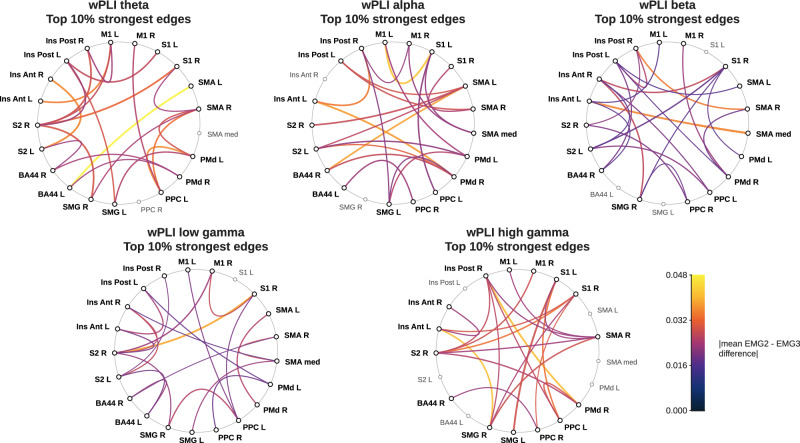
Exploratory whole-network functional connectivity structure during swallowing. Circular plots illustrate exploratory group-average wPLI connectivity differences (EMGswallow – EMGrest) across all 21 cortical ROIs for each frequency band. For visualisation purposes, only the strongest 10% of absolute group-average connectivity differences are displayed. Edge colour and thickness represent relative connectivity magnitude within each frequency band. These visualisations are descriptive summaries of whole-network interaction structure and are not intended for inferential statistical interpretation. Statistically evaluated cluster-corrected subnetworks are shown separately in Fig. 2. Sample size: *n* = 74 participants.

**Fig. 4:**
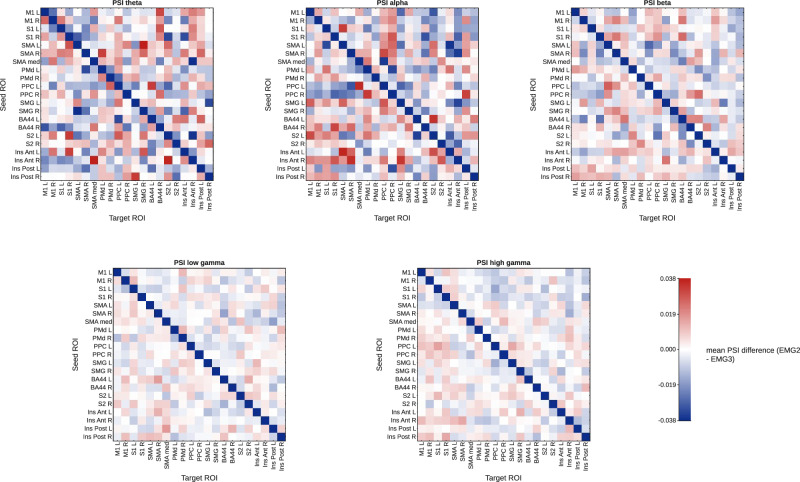
Exploratory whole-network directed connectivity structure during swallowing. ROI×ROI heatmaps illustrate exploratory group-average PSI differences (EMGswallow – EMGrest) across all cortical ROIs for each frequency band. Rows indicate seed ROIs and columns indicate target ROIs. Warmer colours indicate relatively positive PSI differences, whereas cooler colours indicate relatively negative PSI differences. These visualisations are descriptive and do not imply statistically significant directed effects unless explicitly indicated by cluster-corrected analyses in Fig. 2. Sample size: *n* = 74 participants.

To contextualise the cluster-corrected findings, we visualised the strongest 10% of wPLI connections per frequency band based on absolute group-level EMGswallow – EMGrest differences. These exploratory network representations illustrated the distributed spatial organisation of swallowing-related functional connectivity changes across sensorimotor, parietal, and insular regions (Fig. 3). Complementary exploratory ROI×ROI connectivity matrices illustrating mean group-level wPLI differences across all ROI pairs are provided in Supplementary Fig. 1. Exploratory PSI heatmaps illustrate frequency-specific patterns of directed cortical interactions across the swallowing network at the full ROI × ROI matrix level (Fig. 4). These visualisations are descriptive and not intended for inferential interpretation.

### Global network organisation was largely preserved during swallowing

**Fig. 5:**
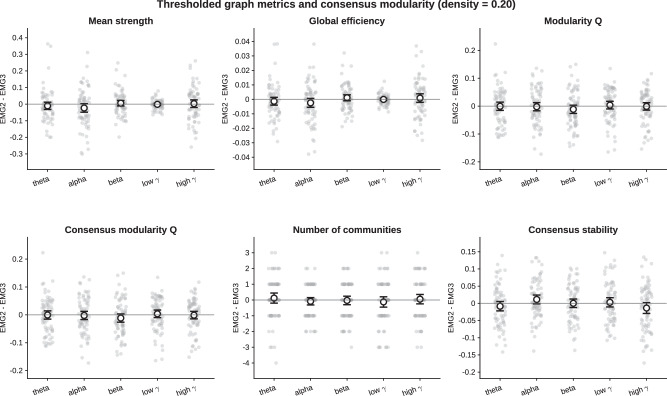
Thresholded graph metrics and consensus modularity. Differences between swallowing conditions (EMGswallow − EMGrest) for thresholded graph metrics (top row: mean strength, global efficiency, modularity Q) and consensus modularity measures (bottom row: consensus modularity Q, number of communities, consensus stability) at a proportional threshold density of 0.20. Individual participant data (*n* = 74) are shown as grey dots. Open circles indicate group means and error bars represent 95% confidence intervals. Positive values indicate higher metric values during EMGswallow (EMG2), whereas negative values indicate higher values during EMGrest (EMG3). Graph metrics were calculated on proportionally thresholded weighted connectivity matrices; consensus modularity measures were derived from repeated Louvain community detection. None of the comparisons remained significant after false discovery rate correction.

We next examined whether focal connectivity changes were accompanied by alterations in large-scale network topology. Graph-theoretical analyses were performed across multiple proportional network densities to reduce dependence on a single threshold selection. None of the examined global metrics, including mean strength, global efficiency, characteristic path length, modularity, and number of communities, differed significantly between swallowing and the control condition after FDR correction (Fig. 5). Repeated Louvain modularity analyses likewise did not reveal significant condition effects for modularity or number of communities across frequency bands. Together, these findings indicate that swallowing-related cortical interactions emerged within an overall stable large-scale network architecture. Complete graph-theoretical metrics and statistical results are provided in Supplementary Data 5.

### Node-level hub organisation remained stable across swallowing and rest

Node-level hub metrics were examined to assess potential changes in regional network centrality during swallowing. Node strength, weighted degree, betweenness centrality, and eigenvector centrality did not reveal FDR-significant condition effects. Exploratory node-level metrics are summarised in Supplementary Fig. 2.

### Quality-control and robustness analyses supported the stability of the connectivity estimates

Quality-control analyses confirmed complete ROI availability and stable connectivity matrix estimation across participants and frequency bands. Additional robustness analyses, including global whole-network wPLI permutation tests and leakage-control analyses based on symmetric orthogonalization, provided no evidence that the main connectivity patterns were driven by nonspecific global shifts in connectivity strength or zero-lag shared signal components (Supplementary Data 6 and 7). Overall, swallowing was associated with frequency-specific and temporally structured cortical interactions involving sensorimotor, parietal, and insular subnetworks embedded within a stable large-scale network architecture.

## Discussion

In this study, we identified focal, frequency-specific, and temporally structured changes in cortical connectivity accompanying voluntary human swallowing. Significant effects involved selective interactions among sensorimotor, parietal, supramarginal, and insular regions and emerged within defined temporal windows around swallow execution. Directed connectivity analyses provided complementary evidence for sparse high-gamma interactions centred on somatosensory and insular regions, while laterality analyses revealed temporally specific rightward asymmetries of directed coupling. Together, these findings indicate that human swallowing is supported by transient and spatially selective cortical interactions that can be resolved at the level of individual sensorimotor acts.

A central finding of the present study is that swallowing-related cortical interactions were anatomically selective and dynamically organised across subsecond timescales. Significant effects involved sensorimotor, premotor, parietal, supramarginal, and insular regions that are consistently implicated in voluntary and reflexive swallowing control^8–10,12,20^. Previous fMRI and effective-connectivity studies suggested that swallowing engages temporally evolving interactions among these regions rather than isolated cortical activations^7,11^. The present findings extend this framework by demonstrating that these interactions can be resolved as transient, frequency-specific subnetworks at the level of individual swallowing acts using source-resolved MEG connectivity analysis.

The frequency specificity of the observed effects suggests that swallowing-related cortical coordination is distributed across multiple oscillatory regimes. This interpretation is consistent with the concept of “spectral fingerprints,” according to which inter-regional neuronal communication is organised through distinct frequency channels with partially separable computational roles^2^. Similar frequency-specific interaction architectures have been described across visual, attentional, and sensorimotor systems^21–24^. Prior MEG studies of swallowing mainly reported alpha- and beta-band modulation in terms of regional oscillatory power changes^10,25,26^. The present analyses extend these findings by examining inter-regional connectivity rather than regional activation alone. Specifically, we identified focal theta-, low-gamma-, and high-gamma-band subnetworks characterised by frequency-specific cortical interactions. This suggests that regional oscillatory activation and inter-regional phase-based connectivity capture complementary aspects of swallowing-related cortical dynamics^22,24,27,28^.

Within this framework, the theta-band wPLI cluster centred on right S2 may reflect coordinated sensory integration within a somatosensory–insular subnetwork involved in monitoring afferent feedback during swallowing^7^. In contrast, the low-gamma wPLI and high-gamma PSI effects may indicate temporally constrained interaction patterns within sensorimotor and insular circuits during swallowing execution, consistent with broader models linking gamma-band synchronisation to temporally precise cortical coordination and sensory integration^21,22^. Because high-frequency MEG connectivity estimates remain more susceptible to muscle contamination and signal-to-noise limitations^29^, the gamma-band findings may be interpreted as evidence for frequency-specific interaction structure rather than direct proof of a specific mechanistic communication mode.

The relationship between the present findings and classical alpha/beta effects on swallowing warrants particular consideration. Alpha rhythms have been associated with functional inhibition and large-scale coordination of cortical processing^30,31^, whereas beta-band activity is closely linked to sensorimotor state regulation and motor control^23,32,33^. Prior swallowing studies primarily reported alpha- and beta-band power modulation^10,25,26^, while the present analyses identified focal theta- and gamma-band connectivity subnetworks. Together, these observations suggest that local oscillatory activation and inter-regional connectivity may reflect complementary aspects of swallowing-related cortical dynamics. Future studies combining spectral and connectivity analyses may further clarify how local oscillatory dynamics relate to transient cortical coordination during swallowing.

Directed connectivity analyses provided additional insight into the temporal organisation of swallowing-related cortical interactions. Significant PSI effects were confined to a small number of anatomically selective high-gamma seed-to-ROI subnetworks centred on the primary somatosensory cortex and the anterior insula. These regions are well-positioned to support sensorimotor integration, somatosensory monitoring, and interoceptive aspects of swallowing control^7,20^. PSI estimates directionality from frequency-dependent phase gradients and has been proposed as a robust approach for assessing directed interactions in electrophysiological data^16^. Within this framework, the observed PSI effects suggest that swallowing-related cortical communication may involve temporally structured and spatially selective directional interactions rather than diffuse large-scale directed coupling. At the same time, directed connectivity derived from MEG reflects statistical asymmetries in oscillatory coupling rather than direct anatomical or mechanistic causality, particularly in the presence of source leakage, linear mixing, and model dependence of connectivity estimates^29,34^. The present PSI findings, therefore, represent evidence for selective frequency-specific asymmetries in cortical interaction structure during swallowing.

The time-resolved findings support the view that swallowing-related cortical interactions evolve dynamically across the behavioural sequence. The most robust temporal effect was low-gamma coupling between the posterior parietal cortex and the posterior insula, with stronger connectivity during later swallowing phases. Posterior parietal regions contribute to multisensory integration and sensorimotor transformation, whereas the insula is central to visceral and interoceptive aspects of bodily state representation^20,35^. Sequential fMRI studies similarly demonstrated that cortical activity and effective connectivity evolve across successive stages of swallowing execution^11^. The present findings extend this temporal perspective to source-level MEG connectivity and suggest that later swallowing phases may involve increased integration of somatosensory, parietal, and interoceptive information.

Laterality analyses revealed rightward theta-band asymmetries of directed interactions during later swallowing phases involving SMA, PMd, S2, and posterior insula. Hemispheric asymmetries are a recurrent feature of cortical swallowing control. Early PET and lesion studies demonstrated asymmetric swallowing representation and suggested that post-stroke dysphagia depends on the integrity of distributed bilateral swallowing networks rather than lesion location alone^8,36^. An MEG study further showed dynamic hemispheric involvement during volitional swallowing, with a temporal shift from earlier left-hemispheric to later right-hemispheric dominance^37^. The present findings align with this temporal framework and extend it from regional activation patterns to directed inter-regional interactions. This suggests that later stages of swallowing may involve rightward-biased coordination among sensorimotor and insular components of the swallowing network.

At the level of global network organisation, graph-theoretical analyses indicated largely preserved whole-network topology across swallowing and rest. Global efficiency, characteristic path length, repeated Louvain-derived modularity estimates, and node-level hub measures did not show robust condition effects after correction for multiple comparisons. This overall stability is consistent with current models of brain network organisation proposing that efficient cortical systems maintain a relatively stable global organisation while flexibly engaging transient task-relevant subnetworks according to behavioural demands^3–5,19^. Within this framework, the present findings suggest that rapid swallowing-related sensorimotor coordination may primarily emerge through selective modulation of anatomically constrained subnetworks rather than through broad reconfiguration of global cortical topology. This interpretation aligns with the temporally focal and frequency-specific connectivity structure observed throughout the present analyses.

Taken together, the present findings support the view that rapid sensorimotor behaviour is accompanied by transient and anatomically selective cortical interactions. Unlike slowly varying resting-state or sustained task-related brain states^1,4,38^, voluntary swallowing provides a model of rapid event-level coordination combining precise physiological event locking, continuous sensory feedback, and tightly constrained motor execution^7,20^. Across the present analyses, swallowing-related cortical interactions were characterised by temporally structured and frequency-specific subnetworks embedded within a comparatively stable global architecture. These findings suggest that rapid sensorimotor behaviour may rely primarily on selective recruitment of transient task-relevant subnetworks rather than broad reconfiguration of whole-network topology.

The clinical implications of this framework may be important. Dysphagia is common across stroke, neurodegenerative disease, and other neurological disorders and is associated with substantial morbidity^39–41^. Contemporary models suggest that swallowing impairment and recovery depend not only on focal lesion location, but also on the integrity and reorganisation of distributed sensorimotor systems^7,11,42^. Within this context, the present data provide a temporally and frequency-resolved reference framework of healthy cortical swallowing interactions.

Several limitations should be considered. Although source-resolved MEG reduces the impact of volume conduction relative to sensor-space analyses, residual source leakage cannot be fully excluded. To mitigate this issue, we employed phase-based connectivity measures that are comparatively insensitive to zero-lag coupling and additionally performed supplementary leakage-control analyses based on symmetric orthogonalization. Nevertheless, some degree of spatial leakage remains an inherent limitation of non-invasive electrophysiological connectivity inference^13,15,17^. Directed connectivity estimates derived from PSI should likewise be interpreted as measures of statistical directionality rather than direct mechanistic causality^16,29^. We focused primarily on cortical interactions, whereas subcortical and brainstem structures constitute critical components of swallowing control but remain difficult to resolve reliably with MEG. In addition, swallowing was examined under controlled experimental conditions in healthy adults; future studies will need to determine whether the observed network organisation generalises to patient populations and more naturalistic swallowing behaviour. Finally, the current study focused on within-frequency interactions, whereas future investigations incorporating cross-frequency coupling may reveal additional coordination mechanisms linking preparatory, sensory, and execution-related processes^43^. Because swallowing is inherently accompanied by oropharyngeal muscle activity, careful artefact control represented an important component of the analytical framework. Contaminated trials were excluded using predefined frequency-specific amplitude, z-score, and temporal-gradient criteria, and analyses were restricted to datasets retaining sufficient valid trials per condition. In addition, source-level LCMV reconstruction and phase-lagged connectivity metrics were used to reduce sensitivity to spatially widespread non-neuronal signal components. Supplementary quality-control analyses supported the robustness of the observed connectivity patterns against nonspecific muscle-related contamination.

In summary, the present study demonstrates that voluntary human swallowing is accompanied by focal, frequency-specific, and temporally structured cortical interactions involving sensorimotor, parietal, supramarginal, and insular subnetworks. By combining precise physiological event locking with source-resolved MEG connectivity analysis, the present work shows that structured cortical interaction dynamics can be resolved at the level of individual sensorimotor acts on subsecond timescales. More broadly, these findings establish voluntary swallowing as a particularly informative model for investigating rapid cortical sensorimotor coordination in humans and provide an event-level framework for studying transient network dysfunction in dysphagia and related neurological disorders.

## Online methods

### Participants

Seventy-five healthy right-handed adults participated (mean age 27.96 ± 6.11 years; 39 females, 36 males). Before participation, individuals were asked about relevant medical history; participants reported no history of neurological, psychiatric, or swallowing disorders and no medication affecting the central nervous system. The study was approved by the Ethics Committee Westfalen-Lippe, and all procedures were conducted in accordance with the Declaration of Helsinki. All participants provided written informed consent before participation.

### Sample size

No a priori power calculation was performed. The sample size was determined by the availability of eligible datasets acquired in prior studies using the same MEG swallowing paradigm and acquisition setup; all participants meeting the inclusion criteria were pooled for the present re-analysis.

### Experimental paradigm and data acquisition

Participants underwent whole-head MEG recording (275-channel Omega 275, CTF Systems Inc.) in a magnetically shielded room. Signals were sampled at 600 Hz. Head position was continuously tracked using localisation coils. During the task, participants performed self-paced volitional swallows at comfortable intervals while keeping the head still. Non-carbonated water was infused continuously into the oral cavity via a thin tube (inner diameter 4.7 mm). The active swallowing interval (EMGswallow) was defined relative to the onset of the pharyngeal swallow phase, as identified from the submental EMG signal (as described by Suntrup et al.^44^). The control interval (EMGrest) comprised a 1-s post-swallow segment beginning approximately 600 ms after pharyngeal swallow onset, ensuring minimal contamination by task-related muscle activity. This procedure matches established paradigms from our group^25,26,44,45^.

### Preprocessing and trial definition

Continuous MEG data were segmented into 1-s epochs. Swallow trials (EMGswallow, EMG2) extended from −0.4 to +0.6s  relative to the peak of submental EMG activity, which was used as an operational marker of pharyngeal swallow onset. Rest trials (EMGrest, EMG3) covered the subsequent 0 to +1 s post-swallow interval (see Fig. 1 for illustration of EMG trace and trial windows). Line noise was attenuated with notch filters at 50, 100, and 150 Hz. A bandpass filter (1–100 Hz) was applied for analyses up to low gamma; for high-gamma analyses, the upper cutoff was extended accordingly. Artefact rejection combined automated and visual inspection. Trials were excluded if they exceeded predefined thresholds for maximum absolute amplitude, maximum absolute z-score, or maximum temporal gradient. For theta, alpha, and beta bands, thresholds were set to an absolute amplitude of 6 × 10⁻¹² T, a maximum |z| of 10, and a temporal gradient of 6 × 10⁻¹² T. For low- and high-gamma bands, more permissive thresholds were applied to account for increased high-frequency muscle contamination, using an absolute amplitude threshold of 1 × 10⁻¹¹ T, a maximum |z| of 15, and a temporal gradient of 1.5 × 10⁻¹¹ T. Trials containing NaNs were discarded, and analyses were restricted to datasets retaining at least five valid trials per condition. For group-level connectivity analyses, we further required ≥8 valid observations per subject–ROI pair. Additional supplementary quality-control analyses supporting the robustness of the preprocessing and artefact-rejection framework are provided in Supplementary Fig. 3.

### Source Analysis and ROI definition

Source reconstruction was performed with a linearly constrained minimum variance (LCMV) beamformer^46^. A standardised single-shell head model derived from the SPM12 template brain and a warped MNI grid with 10-mm spacing were used to ensure consistent source reconstruction and ROI sampling across participants, as individual MRIs were not available for all subjects. The beamformer was configured for fixed orientation and single-trial output, with the covariance matrix estimated over the full 1-s epoch. Twenty-one cortical regions of interest (ROIs) were defined as 10-mm radius spheres centred on established MNI coordinates (Supplementary Table 1), covering bilateral primary motor and somatosensory cortices, premotor areas, inferior frontal gyrus (BA44), supplementary motor area (SMA), inferior parietal and supramarginal gyri, superior temporal gyrus, and anterior and posterior insula. ROIs were selected a priori based on prior neuroimaging and neurophysiological studies implicating these regions in swallowing-related sensorimotor control. ROI time series were extracted as the mean across all grid points within each sphere (see Supplementary Fig. 4 for illustration of ROI locations).

### Connectivity analysis

Spectral estimates were obtained using a multi-taper Fourier transform (DPSS, mtmfft), evaluated at the centre frequency of each band, with frequency smoothing set to half the band width via the multitaper spectral concentration parameter. Phase-based functional connectivity was quantified using the debiased weighted Phase Lag Index (wPLI^15^), which captures consistent non-zero phase-lagged interactions based on the imaginary component of the cross-spectrum. To assess robustness against residual zero-lag source leakage, we performed an additional leakage-control analysis in which ROI time series were symmetrically orthogonalized prior to connectivity estimation^47^. Debiased wPLI was then recomputed across all frequency bands, and global mean orthogonalized connectivity was compared between swallowing and rest conditions using paired statistical testing with FDR correction across frequencies (Supplementary Data 7). This control analysis was intended to evaluate whether global connectivity patterns were robust to aggressive suppression of zero-lag shared signal components; it was not intended to re-estimate the primary cluster-corrected seed-to-ROI effects. Directed interactions were assessed using the Phase Slope Index (PSI^16^), which estimates the relative directionality of oscillatory interactions from the slope of phase differences across neighbouring frequencies, rather than from absolute phase offsets. Directed connectivity metrics have been widely used to estimate statistical directionality within distributed cortical networks^48,49^. Directed connectivity measures derived from MEG capture statistical asymmetries in oscillatory coupling rather than direct causal interactions^29,34^. Connectivity was computed for the full 1-s epoch and for two predefined sub-windows (0–500 ms and 500–1000 ms) to capture early versus late dynamics (see Fig. 1). For higher-frequency bands (beta and gamma), we additionally performed time-resolved connectivity estimation using non-overlapping 250-ms windows, which provide sufficient oscillatory cycles for stable phase-based estimates (e.g., ≥3.25 cycles at 13 Hz and ≥7.5 cycles at 30 Hz). Lower-frequency bands (theta and alpha) were not analysed with 250-ms windows to avoid unreliable phase estimates due to insufficient cycle counts. Multitaper spectral estimation was performed using a frequency-dependent smoothing parameter (tapsmofrq), yielding at least 2 Slepian tapers per window at beta and gamma frequencies, thereby ensuring stable spectral estimates despite short window lengths. Lower-frequency bands (theta and alpha) were analysed using longer time windows to preserve an adequate number of oscillatory cycles per estimate, thereby avoiding unreliable phase estimates at low frequencies^15,29^. Interpretation of lower-frequency time-resolved effects nevertheless remains cautious due to the limited number of oscillatory cycles available within short temporal windows.

### Statistics and reproducibility

Across-participant within-subject inference on connectivity was performed using nonparametric cluster-based permutation testing (CBPT; 5000 permutations, two-sided, α = 0.05, cluster-level corrected), contrasting swallowing (EMGswallow) and rest (EMGrest). CBPT was applied to both ROI-to-ROI and seed-to-ROI connectivity matrices. Graph-theoretical metrics and laterality indices were compared within subjects using paired-sample *t*tests. False discovery rate (FDR) correction was applied within predefined families of comparisons (graph metrics × frequency band; laterality × frequency × time window). Effect sizes (Cohen’s dz) and 95% confidence intervals are reported for all parametric tests. In addition, robustness to linear source leakage was assessed by recomputing debiased wPLI after symmetric orthogonalization of ROI time series (Supplementary Data 7). As an additional global robustness analysis, mean whole-network wPLI was compared between EMGswallow and EMGrest using permutation-based testing across frequency bands. This analysis was intended to assess whether the observed seed-to-ROI findings were accompanied by nonspecific global shifts in connectivity strength, rather than to re-estimate the primary cluster-corrected effects. Results are reported in Supplementary Data 6. Sample size varied across analyses due to the availability of EMG-derived covariates. Connectivity estimates were computed only for subject–ROI pairs with at least eight valid observations, where an observation corresponds to a successfully retained trial contributing to the spectral estimate within the respective condition and frequency band. Group-level summary statistics for global graph-theoretical metrics, including false discovery rate–corrected results, are reported in Supplementary Data 5.

### Laterality analysis

To assess hemispheric asymmetries in cortical connectivity, laterality indices (LI) were computed separately for directed (PSI) and undirected (wPLI) connectivity measures. For directed connectivity, LI values were derived from Phase Slope Index (PSI) estimates according to:

$$L{I}_{\left\{i,j\right\}}={PSI}\left(i\to {j}\right)-{PSI}\left(j\to {i}\right)$$

Positive values indicate relatively stronger left-to-right directed interactions, whereas negative values indicate relatively stronger right-to-left directed interactions.

For undirected connectivity (wPLI), laterality indices were computed between homologous left- and right-hemispheric ROI pairs as:

$$L{I}^{{wPLI}}=\frac{{wPL}{I}_{{Left}}-{wPL}{I}_{{Right}}}{{wPL}{I}_{{Left}}+{wPL}{I}_{{Right}}}$$

where positive values indicate relatively stronger left-hemispheric coupling and negative values indicate relatively stronger right-hemispheric coupling.

Group-level inference was performed using one-sample *t-*tests of LI values against zero, with false discovery rate (FDR) correction applied across ROI pairs within each frequency × time-window block. Analyses included all 21 predefined ROIs (see Supplementary Table 1) and were restricted to subject–ROI pairs with ≥8 valid observations.

### Functional connectivity, seed-to-ROI, and network analyses

Single-trial source time series from the 21 predefined ROIs were used to compute functional (wPLI) and directed (PSI) connectivity for each subject, condition, frequency band, and time window. Connectivity profiles were derived for each ROI treated as a seed against all other ROIs. Task-related effects were assessed using cluster-based permutation testing (CBPT; 5000 permutations, α = 0.05, cluster-corrected), comparing swallowing (EMGswallow) to rest (EMGrest), as well as early (0–500 ms) versus late (500–1000 ms) swallowing phases.

For graph-theoretical analyses, weighted undirected wPLI matrices were analysed using the Brain Connectivity Toolbox^18^. To reduce sensitivity to weak global connectivity fluctuations and reviewer concerns regarding unthresholded weighted graphs, matrices were proportionally thresholded across network densities from 0.10 to 0.30 in steps of 0.05. At each density, the strongest proportion of absolute symmetrized wPLI connections was retained, while all remaining connections were set to zero. Graph metrics were computed separately for each threshold density, and full density-dependent results are reported in Supplementary Data 5. Figure 5 displays density = 0.20 as a representative visualisation.

Global network topology was characterised using mean strength, global efficiency, characteristic path length, modularity Q (Louvain algorithm), and number of communities. For characteristic path length estimation, retained edge weights were converted to connection lengths using the inverse of the connection weights. Because Louvain community detection is stochastic and the present ROI network comprised only 21 nodes, modularity estimation was repeated 100 times for each subject, condition, frequency band, and threshold density. Modularity Q and the number of detected communities were averaged across repetitions to reduce dependence on single stochastic partitions.

Within-subject contrasts between EMGswallow and EMGrest were assessed using paired-sample *t-*tests, with false discovery rate (FDR) correction applied separately within each threshold density across graph metrics and frequency bands. For parametric comparisons, effect sizes are reported as Cohen’s dz with corresponding 95% confidence intervals. Full ROI- and seed-level statistical results are reported in Supplementary Data 1 and 2. Exploratory node-level hub metrics are summarised in Supplementary Fig. 2.

### Ethics statement

The analyses were conducted in accordance with the Declaration of Helsinki and were approved by the Ethics Committee of the Medical Association of Westphalia-Lippe (Ethik-Kommission Westfalen-Lippe; approval no. 2023-142-f-S). All participants provided written informed consent prior to participation.

### Reporting summary

Further information on experimental design is available in the Nature Portfolio Reporting Summary linked to this Article.

## Supplementary information

Transparent Peer Review file

Supplementary Information

Description of Additional Supplementary files

Supplementary Data 1

Supplementary Data 2

Supplementary Data 3

Supplementary Data 4

Supplementary Data 5

Supplementary Data 6

Supplementary Data 7

Reporting Summary

## Data Availability

Cortical networks were visualised using complementary approaches. Seed-to-ROI connectivity was displayed as circular and chord diagrams, while whole-network topology was rendered on inflated cortical surfaces with BrainNet Viewer^50^. Node positions correspond to the 21 predefined ROIs (Supplementary Fig. 4; Supplementary Table 1). Connectivity strength was encoded by edge colour and thickness, and directed interactions were indicated by arrows (Figs. 2–5). Exploratory whole-network visualisations were based on thresholded group-average connectivity differences and are intended for descriptive illustration rather than inferential interpretation. All analyses were implemented in MATLAB using FieldTrip^51^, the Brain Connectivity Toolbox^18^, BrainNet Viewer^50^, and publicly available plotting functions (circularGraph, chordPlot). All custom MATLAB scripts for preprocessing, source analysis, and connectivity estimation are publicly available at [10.5281/zenodo.20393275]. Due to institutional and data-protection regulations applying to high-dimensional neurophysiological datasets, raw MEG recordings are not publicly deposited in an unrestricted repository. De-identified derived datasets and analysis outputs supporting the findings of this study are available from the corresponding author upon reasonable request and subject to institutional data-sharing regulations. Source data underlying the figures are provided in Supplementary Data 1–8. Supplementary Data 5 contains the numerical source data underlying Fig. 5.
