# Supplementary material for: Frequency-specific cortical subnetworks support fast human swallowing
Source: Commun Biol. 2026 Aug 7;9:1059. doi: 10.1038/s42003-026-10751-6 (PMC13451196; doi:10.1038/s42003-026-10751-6)
Supplement: Supplementary file 2 [file 42003_2026_10751_MOESM1_ESM.pdf]

nature portfolio

Peer Review File

Frequency-specific cortical subnetworks support fast human swallowing

Corresponding Author: Dr Paul Muhle

This file contains all reviewer reports in order by version, followed by all author rebuttals in order by version.

Attachments originally included by the reviewers as part of their assessment can be found at the end of this file.

Version 0:

Reviewer comments:

Reviewer #1

(Remarks to the Author)
Overview
This article establishes aims to target a very intriguing function – swallowing which is of practical importance in neurological disorders. The approach attempted is in-vivo MEG in humans and identification of a functional network motif associated with sub second sensorimotor coordination time locked to swallowing events. MEG was collected from a large sample of 74 subjects during continuous volitional swallowing which is very decent. Resting state and swallowing epochs were identified by using EMG recording to identify the onset of swallowing. Phase locking measures and graph theory were used to identify interacting regions and overall topology changes associated with fast sensorimotor coordination. While these are some of the strong suits there are some fundamental flaws with the manuscript

Issues
Major

The manuscript severely lacks a demonstrative aspect of how the raw data looks like. How does a whole-brain representation of the swallowing evoked field be distributed in terms of evoked/induced power? Without this any further analysis cannot be interpreted.

Since the authors are relying on ICA methods, how do the muscle related noise gets removed in MEG? This is an important question, because every result from the network analysis such as entropy/efficency could simply be explained by increase in common noise introduced by muscle movement either by the act or due to the planning related components.

The directed connectivity analysis, based on the PSI and cluster based permutation testing results in the survival of only a single cluster in the beta band. This is used as evidence of ‘selective’ directed interactions. However, considering the fact that the split half reliability of PSI in the beta band is close to 0 (See Supplementary figure S3) this result is overstated. The low reliability of PSI in the relevant frequency band is essential context for this result and should be explicitly stated in the main text.

The authors use functional connectivity matrices to calculate global efficiency and characteristic path length by converting connection strength to path length. However, the wPLI values used to construct the FC matrix are unthresholded. As a result, small, global changes to connection weight caused by noise could change the value of these measures. To distinguish this from genuine network integration, thresholding is recommended prior to performing this analysis.

The modularity analysis is based on the Louvain community detection algorithm; however, the graph only has 21 nodes. To ensure the communities are robust to the stochasticity of this algorithm, consensus clustering could be applied.
Minor
The introduction (line 60) states,

‘... It remains poorly understood how cortical networks are transiently configured to support fast sensorimotor acts …’

Which implies that the paper will address the mechanism of transient synchronization for fast sensorimotor actions. This is misleading considering the descriptive nature of the results to follow. Being that the results are novel and describe an unresolved phenomenon, an alternative framing emphasizing this would be both compelling and faithful to the work.

Near zero connectivity estimates following symmetric orthogonalization are used to rule out signal leakage as an explanation for the wPLI effects (See Supplementary figure S4). However, the aggressive orthogonalization removes both conduction artefacts and genuine coupling from the signal. This makes the result uninformative regarding the extent of conduction artefacts in the non-orthogonalized signal. Using a more sparing orthogonalization scheme would be appropriate.
Please report exact number of males and females rather than percentages.

Ethics Related
Name of local ethics committee needs to be mentioned. Does the work really abide Declaration of Helsinki which requires dissemination of de-identified data in public data bases?
Data related
Raw data or preprocessed EEG data couldn't be located. Please identify them with appropriate names. Presentation of only meta-data like phase-lagged index matrices are not acceptable in isolation.

Reviewer #2

(Remarks to the Author)
I co-reviewed this manuscript with one of the reviewers who provided the listed reports. This is part of the Communications Biology initiative to facilitate training in peer review and to provide appropriate recognition for Early Career Researchers who co-review manuscripts.

Reviewer #3

(Remarks to the Author)
The overall experimental design is interesting, particularly the exploration of transient changes in cortical networks during swallowing using MEG. However, the presentation of the results is not well organized, making it difficult to comprehend the main conclusions. The figures in the Results section are not clearly presented, and sufficient statistical evidence is lacking to support the reported findings. Thus, the manuscript requires substantial revision before it can be further considered.

In Figure 2, the reported wPLI values are extremely low (maximum around 0.04). It is unclear how such small values can be interpreted as meaningful or significant. Across different frequency bands, each ROI appears to be connected with many others, with both relatively high and low wPLI values. This gives the impression that nearly all regions are connected to each other. Are there specific connections that are statistically stronger than others? Furthermore, Figure 2 lacks appropriate control conditions. It would be important to include comparisons with control data, such as surrogate data or a resting-state baseline. Demonstrating that task-related connectivity exceeds these control conditions would strengthen claims of significance. As currently presented, it is difficult to determine which nodes (regions) are significantly engaged during the task. Although network engagement appears frequency-dependent, it remains unclear which frequencies and regions are most relevant, as nearly all regions show changes without clear prioritization.

In Figure 3, the rationale for selecting specific regions is not explained. Were these chosen solely for illustration purposes? Additionally, the y-axis values in Figures 3D and 3E appear to be more than ten times higher than those in Figures 3A–C. Please clarify whether these values are accurate. While transient changes are visually apparent, such observations do not imply statistical differences without supporting statistical analyses.

Figure 4 cannot be properly evaluated because it appears to be identical to Figure 2. This should be corrected.

In Figure 5, the names of the significant ROIs should be clearly labeled within the figure for clarity.

Figure 6 shows only very minor differences in Global Efficiency and Path Length. Are these differences truly significant, and if so, are they meaningful? In contrast, Modularity appears to show a more substantial difference. The reported p-values for Efficiency and Path Length may be driven by the large sample size (n = 74 subjects), but the effect sizes appear minimal. The statistical tests used should be clearly specified (e.g., paired t-tests), and it should be stated whether multiple-comparison corrections such as Bonferroni correction were applied.

In lines 386–387 and elsewhere, the term "group-level inference" is used. This phrasing suggests comparisons between distinct groups, whereas the study appears to involve different conditions within the same participants. It would be more accurate to specify "group-level inference using a within-subject design" or similar wording.

The manuscript presents an overwhelming amount of statistical information—close to raw statistical outputs—without prioritization, making it challenging for readers to discern which results constitute the main findings. Presenting summary tables with the most important statistical outcomes would greatly improve clarity. The authors should explicitly state which findings are statistically significant and supported by the analyses.

Finally, the time-series analyses use two short windows (–500 to 0 ms and 0 to 500 ms). These brief durations (500 ms each) may not be appropriate for measures such as wPLI, PSI, and graph-theory metrics, particularly in lower frequency

bands. With such short windows, only a few oscillatory cycles are available, making the measures highly sensitive to noise. Even minor disruptions can substantially alter the estimated connectivity metrics. This limitation should be addressed and justified.

Reviewer #4

(Remarks to the Author)
The authors report an extensive, precise, and complete study on the network configuration of cortical activity during swallowing, using MEG. The research question is well posed and relevant to the field of voluntary fast sensorimotor acts and beyond. Moreover, identifying and characterizing network-level activity in different frequency bands has clinical relevance, as it can be used as a biomarker of recovery after sensorimotor impairing diseases, such as stroke in the chronic phase. The study is excellent and complete, and it provides a broad picture of network brain activity during swallowing. The sample size is large (N = 74), ensuring the robustness of the results. Methodological issues are very well addressed and reported in detail. In summary, I strongly suggest the paper for publication in Communications Biology. I do not have further comments, apart from one suggestion. On lines 257–259, where the authors correctly state that “Clinical outcome is often not fully explained by focal lesion location alone; instead, accumulating evidence suggests that impairment and recovery depend on the integrity and reorganization of distributed sensorimotor networks”, I would suggest that they also include references to more recent works on biomarkers of recovery at the network level, such as Brancaccio et al. 2025 (https://doi.org/10.1016/j.clinph.2025.2110747) and/or references therein.

Version 1:

Reviewer comments:

Reviewer #3

(Remarks to the Author)
None.

Reviewer #4

(Remarks to the Author)
The authors carefully tackled all the issues raised by comment, resulting in a strongly improved contribution, both from the methodological and scientific point of view. I do not have further comments and I recommend the paper for publication in Communications Biology.

Open Access This Peer Review File is licensed under a Creative Commons Attribution 4.0 International License, which permits use, sharing, adaptation, distribution and reproduction in any medium or format, as long as you give appropriate credit to the original author(s) and the source, provide a link to the Creative Commons license, and indicate if changes were made.

In cases where reviewers are anonymous, credit should be given to 'Anonymous Referee' and the source.
The images or other third party material in this Peer Review File are included in the article's Creative Commons license, unless indicated otherwise in a credit line to the material. If material is not included in the article's Creative Commons license and your intended use is not permitted by statutory regulation or exceeds the permitted use, you will need to obtain permission directly from the copyright holder.
To view a copy of this license, visit https://creativecommons.org/licenses/by/4.0/

Rebuttal Letter

Frequency-specific cortical subnetworks support fast human swallowing

Formerly *“A temporally orchestrated cortical network state for fast human swallowing”*

We thank the reviewers and the editor for their careful and constructive evaluation of our manuscript. The comments were highly valuable and helped us substantially improve the study’s analytical focus, statistical organization, and interpretational clarity.

During revision, we identified an inconsistency in the originally implemented bilateral PMd ROI coordinates. Because PMd-related connectivity contributed to several downstream analyses, including seed-to-ROI connectivity, laterality analyses, and graph-theoretical measures, we decided to recompute the complete analysis pipeline after correcting the ROI definitions rather than applying localized corrections. This included source-level extraction, connectivity estimation, cluster-based permutation testing, graph-theoretical analyses, laterality analyses, and robustness analyses.

Importantly, the revised analyses led to a more selective and statistically constrained pattern of findings. Compared with the original submission, the revised manuscript now places stronger emphasis on focal, frequency-specific, and temporally structured subnetworks, while large-scale graph-theoretical effects are interpreted substantially more cautiously. Several findings previously discussed more broadly are now presented as exploratory or no longer emphasized after re-analysis.

The manuscript was reorganized accordingly. Cluster-corrected primary findings, exploratory whole-network visualizations, graph-theoretical analyses, and robustness analyses are now more clearly separated throughout the Results and Supplementary Material. Directed connectivity findings are interpreted more cautiously, and additional robustness and leakage-control analyses were added. All figures were updated and are provided in the revised manuscript and Supplementary Information.

Importantly, the revised manuscript intentionally adopts a substantially more conservative interpretational framework than the original submission, focusing on anatomically selective and statistically supported subnetworks rather than large-scale transient network states.

We believe that these revisions substantially strengthened the manuscript conceptually and methodologically and resulted in a clearer interpretation of transient cortical network dynamics during swallowing.

Reviewer #1

We sincerely thank the reviewer for the detailed and thoughtful evaluation of our work. We appreciate the recognition of the large sample size, the event-locked MEG approach, and the focus on fast sensorimotor coordination during swallowing. The reviewer's comments substantially improved the rigor, clarity, and interpretational precision of the revised manuscript.

Comment 1: The manuscript severely lacks a demonstrative aspect of how the raw data looks like. How does a whole-brain representation of the swallowing evoked field be distributed in terms of evoked/induced power? Without this any further analysis cannot be interpreted.

Response 1: We appreciate this important point. In the revised manuscript, we substantially expanded the visualization and quality-control sections to provide a clearer representation of the underlying data and preprocessing workflow. For this, please see Supplementary Figure S3, which now provides additional quality-control analyses including the event-locked EMG-defined trial structure, sensor-level MEG signal structure, induced time–frequency power changes, and an auxiliary-channel control analysis addressing potential muscle-related contamination.

Importantly, the revised manuscript now places substantially stronger emphasis on source-level and inferentially validated connectivity effects rather than broad descriptive whole-network interpretations.

We agree that event-related spectral power constitutes an additional informative physiological level. However, the primary aim of the present study was to characterize event-locked cortical interaction structure rather than evoked-field or power dynamics per se. We have now clarified this distinction explicitly in the revised Introduction and Discussion.

Comment 2: Since the authors are relying on ICA methods, how do the muscle related noise gets removed in MEG? This is an important question, because every result from the network analysis such as entropy/efficency could simply be explained by increase in common noise introduced by muscle movement either by the act or due to the planning related components.

Response 2: We thank the reviewer for this important point. Because swallowing is inherently accompanied by oropharyngeal muscle activity, artifact control was treated as a central component of the revised analysis pipeline. We clarified in the manuscript that

the analysis did not primarily rely on IC-based artifact removal, but combined multiple complementary strategies, including automated frequency-specific trial rejection, exclusion of contaminated trials, source-level LCMV beamforming, and phase-lagged connectivity measures designed to reduce sensitivity to zero-lag common signal components.

In addition, we added supplementary signal-quality analyses showing event-locked sensor-level MEG signal structure and induced power changes during swallowing, as well as additional leakage-control analyses. Importantly, global graph-theoretical measures did not show widespread increases in connectivity after correction for multiple comparisons, arguing against a nonspecific global increase in common noise as the primary explanation of the observed effects.

We also revised the Discussion to explicitly acknowledge that high-frequency MEG connectivity estimates remain susceptible to muscle contamination and should therefore be interpreted cautiously.

In addition, we substantially expanded the robustness analyses. The revised manuscript now includes supplementary leakage-control analyses based on symmetric orthogonalization, global whole-network wPLI permutation analyses, and extensive QC summaries in Supplementary Figure S3 and Supplementary Tables S5 and S6.

Comment 3: The directed connectivity analysis, based on the PSI and cluster based permutation testing results in the survival of only a single cluster in the beta band. This is used as evidence of ‘selective’ directed interactions. However, considering the fact that the split half reliability of PSI in the beta band is close to 0 (See Supplementary figure S3) this result is overstated. The low reliability of PSI in the relevant frequency band is essential context for this result and should be explicitly stated in the main text.

Response 3: We thank the reviewer for this important observation and agree that the low split-half reliability of PSI in the beta band requires careful interpretation. In the revised manuscript, we substantially toned down the interpretation of directed connectivity findings and explicitly state in the main text that PSI-derived directionality should be interpreted cautiously, particularly in frequency bands showing limited reliability.

Importantly, after re-analysis of the dataset following correction of the PMd ROI coordinates, the revised PSI results no longer emphasize broad beta-band directional effects. Instead, the surviving directed interactions were spatially sparse and primarily confined to selective high-gamma subnetworks. We now frame these findings as evidence for anatomically selective and temporally structured directional organization rather than as a strong or global directional hierarchy.

Comment 4: The authors use functional connectivity matrices to calculate global efficiency and characteristic path length by converting connection strength to path length. However, the wPLI values used to construct the FC matrix are unthresholded. As a result, small, global changes to connection weight caused by noise could change the value of

these measures. To distinguish this from genuine network integration, thresholding is recommended prior to performing this analysis.

Response 4: We thank the reviewer for this important methodological point. In the revised manuscript, we substantially softened the interpretation of global graph-theoretical findings and now explicitly acknowledge that weighted network metrics derived from unthresholded connectivity matrices can be influenced by small global changes in connection strength and noise structure.

Importantly, the revised analyses did not reveal robust condition effects in global efficiency or characteristic path length after correction for multiple comparisons. We therefore no longer interpret these measures as evidence for strong global network integration changes during swallowing. Instead, the revised Discussion emphasizes that swallowing-related effects were primarily confined to temporally and anatomically selective subnetworks embedded within a comparatively stable global topology.

In addition, we added threshold-density robustness analyses for graph metrics, repeated Louvain modularity estimation, and leakage-control analyses, to reduce the likelihood that the reported findings reflect nonspecific global shifts in connectivity weight structure.

Comment 5: The modularity analysis is based on the Louvain community detection algorithm; however, the graph only has 21 nodes. To ensure the communities are robust to the stochasticity of this algorithm, consensus clustering could be applied.

Response 5: We thank the reviewer for this valuable suggestion. In response, we revised the graph-theoretical analyses to reduce sensitivity to stochastic variation in Louvain-based modularity estimation within the relatively small 21-node ROI network.

Specifically, graph metrics were recomputed after proportional thresholding of weighted wPLI matrices across multiple network densities (10–30%). For modularity analyses, the Louvain algorithm was repeated 100 times for each subject, condition, frequency band, and threshold density. Modularity Q and the number of detected communities were then averaged across repetitions to reduce dependence on single stochastic community partitions.

Importantly, the revised analyses did not reveal robust condition-dependent modularity differences after correction for multiple comparisons. We therefore substantially toned down the interpretation of global graph-theoretical effects and now emphasize that swallowing-related effects were primarily expressed as anatomically selective and temporally structured subnetworks embedded within a comparatively stable global topology.

Comment 6: The introduction (line 60) states,

‘... It remains poorly understood how cortical networks are transiently configured to support fast sensorimotor acts …’

Which implies that the paper will address the mechanism of transient synchronization for fast sensorimotor actions. This is misleading considering the descriptive nature of the results to follow. Being that the results are novel and describe an unresolved phenomenon, an alternative framing emphasizing this would be both compelling and faithful to the work.

Response 6: We thank the reviewer for this important conceptual point. We agree that the original wording of the Introduction could be interpreted as implying a mechanistic explanation of transient synchronization processes. We therefore revised the framing throughout the Introduction to more clearly emphasize the descriptive and characterization-oriented nature of the present work. Specifically, the revised text now focuses on the identification and characterization of transient cortical interaction patterns during rapid sensorimotor behaviour rather than implying direct mechanistic inference.

Comment 7: Near zero connectivity estimates following symmetric orthogonalization are used to rule out signal leakage as an explanation for the wPLI effects (See Supplementary figure S4). However, the aggressive orthogonalization removes both conduction artefacts and genuine coupling from the signal. This makes the result uninformative regarding the extent of conduction artefacts in the non-orthogonalized signal. Using a more sparing orthogonalization scheme would be appropriate.

Response 7: We thank the reviewer for this important methodological point and agree that symmetric orthogonalization represents a highly conservative leakage-control procedure that may suppress both spurious zero-lag signal components and physiologically meaningful coupling. We therefore revised the manuscript to avoid interpreting the near-zero connectivity estimates after orthogonalization as evidence that source leakage can be excluded from the non-orthogonalized data.

Instead, the orthogonalization analysis is now presented as a supplementary sensitivity analysis demonstrating that the reported connectivity structure is not trivially preserved under aggressive leakage suppression. We explicitly acknowledge in the revised Discussion that source leakage remains an inherent limitation of non-invasive MEG connectivity analysis despite the use of phase-lagged metrics and source reconstruction approaches.

Comment 8: Please report exact number of males and females rather than percentages.

Response 8: We thank the reviewer for noting this point. The exact numbers of female and male participants are now reported in the Methods section instead of percentages.

Comment 9: Ethics Related – Name of local ethics committee needs to be mentioned. Does the work really abide Declaration of Helsinki which requires dissemination of de-identified data in public data bases?

Response 9: We thank the reviewer for this important point. The name of the local ethics committee (ethic committee Westfalen-Lippe) has now been explicitly added to the Methods section together with the corresponding approval information.

All procedures were conducted in accordance with the Declaration of Helsinki, and all participants provided written informed consent prior to participation. Due to the sensitive nature of raw MEG datasets and institutional data-protection regulations, fully anonymized raw data are not publicly deposited in an unrestricted repository. However, de-identified derived data and analysis outputs relevant to the present study are available from the corresponding author upon reasonable request and in accordance with local ethics and data-protection requirements.

We additionally revised the Data Availability statement to clarify which processed datasets and analysis materials are accessible.

Comment 10: Data related – Raw data or preprocessed EEG data couldn't be located. Please identify them with appropriate names. Presentation of only meta-data like phase-lagged index matrices are not acceptable in isolation.

Response 10: We thank the reviewer for this important point. We agree that connectivity matrices alone are insufficient to fully contextualize the underlying neurophysiological signal structure. In response, we added supplementary signal-quality analyses demonstrating event-locked sensor-level MEG responses, induced time–frequency power modulation, and additional artifact-control analyses associated with swallowing-related activity.

We also revised the Data Availability statement to more clearly specify which data types are available. The study is based on MEG recordings rather than EEG data. Due to institutional ethics and data-protection regulations applying to high-dimensional neurophysiological datasets, raw MEG recordings are not deposited in an unrestricted public repository. However, de-identified derived datasets, connectivity matrices, supplementary signal-quality analyses, and analysis scripts relevant to the present study are available from the corresponding author upon reasonable request and in accordance with institutional data-sharing regulations.

Reviewer #2

We thank Reviewer #2 for their thoughtful contribution to the review process and for the careful assessment of the manuscript within the Communications Biology co-review initiative supporting Early Career Researchers.

Reviewer #3

We sincerely thank the reviewer for the careful and constructive assessment. We agree that the original manuscript contained an overly broad narrative relative to the inferential strength of the statistical results, and we substantially revised the manuscript accordingly.

Comment 1: The overall experimental design is interesting, particularly the exploration of transient changes in cortical networks during swallowing using MEG. However, the presentation of the results is not well organized, making it difficult to comprehend the main conclusions. The figures in the Results section are not clearly presented, and sufficient statistical evidence is lacking to support the reported findings. Thus, the manuscript requires substantial revision before it can be further considered.

Response 1: We thank the reviewer for the careful evaluation of our manuscript and for highlighting issues regarding clarity, figure presentation, and prioritization of the main findings. In response, we substantially revised the manuscript with the goal of improving readability, interpretability, and statistical transparency throughout.

The Results section was extensively reorganized to provide a clearer progression from focal connectivity findings to directed interactions, temporal effects, laterality analyses, and global network topology. We also substantially revised the Discussion to better distinguish primary findings from exploratory observations and to improve the physiological interpretation of the results.

In addition, multiple figures were redesigned for clarity, including revised labeling, improved panel organization, clearer ROI annotations, and expanded supplementary material. We further added supplementary signal-quality analyses demonstrating event-locked MEG responses and induced power structure underlying the connectivity analyses.

Statistical reporting was also revised throughout the manuscript. Cluster-corrected findings, effect interpretation, correction procedures, and robustness analyses are now more explicitly described, while non-significant or weak effects are interpreted more cautiously. Together, these revisions substantially improved the clarity and organization of the manuscript.

Comment 2: In Figure 2, the reported wPLI values are extremely low (maximum around 0.04). It is unclear how such small values can be interpreted as meaningful or significant. Across different frequency bands, each ROI appears to be connected with many others, with both relatively high and low wPLI values. This gives the impression that nearly all regions are connected to each other. Are there specific connections that are statistically stronger than others? Furthermore, Figure 2 lacks appropriate control conditions. It would be important to include comparisons with control data, such as surrogate data or a resting-state baseline. Demonstrating that task-related connectivity exceeds these control conditions would strengthen claims of significance. As currently presented, it is difficult to determine which nodes (regions) are significantly engaged during the task. Although network engagement appears frequency-dependent, it remains unclear which frequencies

and regions are most relevant, as nearly all regions show changes without clear prioritization.

Response 2: We thank the reviewer for this important comment. We agree that the original presentation of the connectivity matrices did not sufficiently distinguish between descriptive connectivity structure and statistically supported task-related effects.

In the revised manuscript, we substantially reorganized both the figure presentation and the interpretation of the connectivity results. Importantly, the primary statistical inference in this study is not based on the absolute magnitude of individual wPLI values, but on cluster-corrected within-subject contrasts between swallowing (EMG2) and resting/post-swallow control epochs (EMG3). We now emphasize this distinction more clearly throughout the Results and Discussion.

We further clarified that the interpretation of the present connectivity analyses is based primarily on statistically evaluated within-subject contrasts and cluster-corrected subnetwork structure rather than on the absolute magnitude of individual wPLI values alone. The revised manuscript therefore places greater emphasis on anatomically and temporally selective interaction patterns that survived group-level statistical testing.

In response to the reviewer's concern regarding control conditions, we revised the figures and text to more explicitly highlight the EMG3 resting/post-swallow comparison condition underlying the statistical analyses. We additionally added supplementary signal-quality analyses demonstrating event-locked MEG responses and induced power structure associated with swallowing.

We additionally performed an exploratory surrogate-control analysis for beta-band full-window EMGswallow data. For each participant, ROI time series were phase-randomized 100 times while preserving the Fourier magnitude spectrum. Mean connectivity from the original wPLI matrices was then compared with connectivity estimated from phase-randomized surrogate time series. This analysis was intended as a global sanity check against nonspecific phase structure rather than as a replacement for the primary cluster-corrected within-subject inference. Together with the EMGrest control condition and cluster-corrected statistics, this analysis supports the interpretation that the reported effects are not reducible to arbitrary low-amplitude connectivity fluctuations.

Finally, the revised manuscript now places substantially greater emphasis on the specific cluster-corrected subnetworks that survived statistical testing, while global or weak effects are interpreted more cautiously. This revision was accompanied by extensive re-analysis of the dataset following correction of the bilateral PMd ROI coordinates, which resulted in a substantially refined and more anatomically selective connectivity pattern throughout the manuscript.

Comment 3: In Figure 3, the rationale for selecting specific regions is not explained. Were these chosen solely for illustration purposes? Additionally, the y-axis values in Figures 3D and 3E appear to be more than ten times higher than those in Figures 3A–C. Please clarify whether these values are accurate. While transient changes are visually apparent, such observations do not imply statistical differences without supporting statistical analyses.

Response 3: We thank the reviewer for these important comments. In response, we substantially revised and reorganized the entire figure set following re-analysis of the dataset after correction of the bilateral PMd ROI coordinates. As a result, the revised manuscript now presents a substantially refined and more anatomically selective connectivity pattern together with a clearer statistical prioritization of the main findings.

The revised figures and accompanying Results text now explicitly distinguish descriptive visualization from statistically supported effects and more clearly indicate how displayed subnetworks relate to the underlying cluster-corrected analyses. We additionally improved figure labeling, panel organization, ROI annotation, and statistical reporting throughout to improve interpretability and reduce ambiguity regarding the physiological relevance of the displayed effects.

Comment 4: Figure 4 cannot be properly evaluated because it appears to be identical to Figure 2. This should be corrected.

Response 4: We thank the reviewer for noting this issue. Following the full re-analysis and redesign of the figure set, this problem has been corrected. The revised manuscript now clearly separates cluster-corrected subnetwork effects from exploratory whole-network visualizations, and the corresponding figures have been regenerated with distinct content, labeling, and figure legends.

Comment 5: In Figure 5, the names of the significant ROIs should be clearly labeled within the figure for clarity.

Response 5: We thank the reviewer for this helpful suggestion. In the revised manuscript, the corresponding figures were redesigned and significant ROIs are now explicitly labeled within the figure panels and figure legends to improve interpretability and anatomical clarity.

Comment 6: Figure 6 shows only very minor differences in Global Efficiency and Path Length. Are these differences truly significant, and if so, are they meaningful? In contrast, Modularity appears to show a more substantial difference. The reported p-values for Efficiency and Path Length may be driven by the large sample size (n = 74 subjects), but the effect sizes appear minimal. The statistical tests used should be clearly specified (e.g., paired t-tests), and it should be stated whether multiple-comparison corrections such as Bonferroni correction were applied.

Response 6: We thank the reviewer for this important point. We agree that the originally presented global graph-theoretical effects required more cautious interpretation. In the revised manuscript, we substantially toned down the interpretation of global efficiency and characteristic path length findings and now explicitly emphasize that effect sizes were small and did not indicate broad global network reconfiguration during swallowing.

Following re-analysis of the dataset after correction of the bilateral PMd ROI coordinates, the revised analyses no longer support strong conclusions regarding global topology changes. Instead, the manuscript now focuses primarily on anatomically selective and temporally structured subnetwork-level effects.

We additionally clarified the statistical procedures throughout the Methods and Results sections, including the use of within-subject statistical testing and correction for multiple comparisons. The revised manuscript now explicitly distinguishes exploratory graph-level observations from robust cluster-corrected connectivity findings.

Comment 7: In lines 386–387 and elsewhere, the term "group-level inference" is used. This phrasing suggests comparisons between distinct groups, whereas the study appears to involve different conditions within the same participants. It would be more accurate to specify "group-level inference using a within-subject design" or similar wording.

Response 7: We thank the reviewer for this helpful clarification. We agree that the original wording could be misinterpreted as referring to comparisons between independent participant groups. In the revised manuscript, we therefore replaced formulations such as "group-level inference" with more precise wording specifying within-subject statistical inference across experimental conditions where appropriate.

Comment 8: The manuscript presents an overwhelming amount of statistical information—close to raw statistical outputs—without prioritization, making it challenging for readers to discern which results constitute the main findings. Presenting summary tables with the most important statistical outcomes would greatly improve clarity. The authors should explicitly state which findings are statistically significant and supported by the analyses.

Response 8: We thank the reviewer for this important observation. We agree that the original manuscript contained an overly dense presentation of statistical output, which reduced the visibility of the principal findings. In response, we substantially reorganized the Results and Discussion sections to more clearly distinguish primary statistically supported findings from exploratory or descriptive observations.

The revised manuscript now prioritizes cluster-corrected effects, anatomically selective subnetworks, and temporally structured interactions that survived group-level within-subject statistical testing. Non-significant or weak findings are interpreted more cautiously and no longer emphasized to the same extent as statistically robust effects.

In addition, we added summary tables and revised figure organization and labeling throughout the manuscript to improve readability and clarify which findings are directly supported by the statistical analyses.

Comment 9: Finally, the time-series analyses use two short windows (−500 to 0 ms and 0 to 500 ms). These brief durations (500 ms each) may not be appropriate for measures such as wPLI, PSI, and graph-theory metrics, particularly in lower frequency bands. With

such short windows, only a few oscillatory cycles are available, making the measures highly sensitive to noise. Even minor disruptions can substantially alter the estimated connectivity metrics. This limitation should be addressed and justified.

Response 9: We thank the reviewer for this important methodological point. We agree that short analysis windows represent an inherent challenge for oscillatory connectivity estimation, particularly at lower frequencies where fewer oscillatory cycles are available. In the revised manuscript, we therefore explicitly discuss this limitation and interpret lower-frequency time-resolved findings with appropriate caution.

Several aspects of the present design partially mitigate this concern. First, the analyses were performed at the group level across a comparatively large cohort (N = 74), reducing the likelihood that isolated noisy estimates would systematically drive the reported effects. Second, statistical inference relied on within-subject contrasts and cluster-based permutation testing rather than on isolated single-edge estimates. Third, the strongest and most consistent temporal effects emerged in low-gamma and high-gamma ranges, where substantially more oscillatory cycles are present within the analysed windows.

We additionally revised the manuscript to more clearly distinguish robust cluster-corrected findings from exploratory observations and now discuss temporal and low-frequency connectivity effects more cautiously throughout the Discussion and Limitations sections.

Reviewer #4

We sincerely thank the reviewer for the highly positive and encouraging assessment of our work and for recognizing both the methodological depth and the potential clinical relevance of the study.

Comment: The authors report an extensive, precise, and complete study on the network configuration of cortical activity during swallowing, using MEG. The research question is well posed and relevant to the field of voluntary fast sensorimotor acts and beyond. Moreover, identifying and characterizing network-level activity in different frequency bands has clinical relevance, as it can be used as a biomarker of recovery after sensorimotor impairing diseases, such as stroke in the chronic phase. The study is excellent and complete, and it provides a broad picture of network brain activity during swallowing. The sample size is large (N = 74), ensuring the robustness of the results. Methodological issues are very well addressed and reported in detail. In summary, I strongly suggest the paper for publication in Communications Biology. I do not have further comments, apart from one suggestion. On lines 257–259, where the authors correctly state that “Clinical outcome is often not fully explained by focal lesion location alone; instead, accumulating evidence suggests that impairment and recovery depend on the integrity and reorganization of distributed sensorimotor networks”, I would suggest that they also include references to more recent works on biomarkers of recovery at the network level, such as Brancaccio et al. 2025 (https://doi.org/10.1016/j.clinph.2025.2110747) and/or references therein.

Response: In response to the reviewer's helpful suggestion, we expanded the Discussion section to include more recent literature on network-level biomarkers of recovery and distributed sensorimotor reorganization in dysphagia and related neurological disorders. Specifically, we incorporated the suggested work by Brancaccio et al. 2025 together with the surrounding network-neuroscience context to strengthen the translational interpretation of the present findings.

Overview

This article establishes aims to target a very intriguing function – swallowing which is of practical importance in neurological disorders. The approach attempted is in-vivo MEG in humans and identification of a functional network motif associated with sub second sensorimotor coordination time locked to swallowing events. MEG was collected from a large sample of 74 subjects during continuous volitional swallowing which is very decent. Resting state and swallowing epochs were identified by using EMG recording to identify the onset of swallowing. Phase locking measures and graph theory were used to identify interacting regions and overall topology changes associated with fast sensorimotor coordination. While these are some of the strong suits there are some fundamental flaws with the manuscript

Issues

Major

1. The manuscript severely lacks a demonstrative aspect of how the raw data looks like. How does a whole-brain representation of the swallowing evoked field be distributed in terms of evoked/induced power? Without this any further analysis cannot be interpreted.

2. Since the authors are relying on ICA methods, how do the muscle related noise gets removed in MEG? This is an important question, because every result from the network analysis such as entropy/efficency could simply be explained by increase in common noise introduced by muscle movement either by the act or due to the planning related components.

3. The directed connectivity analysis, based on the PSI and cluster based permutation testing results in the survival of only a single cluster in the beta band. This is used as evidence of 'selective' directed interactions. However, considering the fact that the split half reliability of PSI in the beta band is close to 0 (See Supplementary figure S3) this result is overstated. The low reliability of PSI in the relevant frequency band is essential context for this result and should be explicitly stated in the main text.

4. The authors use functional connectivity matrices to calculate global efficiency and characteristic path length by converting connection strength to path length. However, the wPLI values used to construct the FC matrix are unthresholded. As a result, small, global changes to connection weight caused by noise could change the value of these measures. To distinguish this from genuine network integration, thresholding is recommended prior to performing this analysis.

5. The modularity analysis is based on the Louvain community detection algorithm; however, the graph only has 21 nodes. To ensure the communities are robust to the stochasticity of this algorithm, consensus clustering could be applied.

Minor

1. The introduction (line 60) states,

    '... It remains poorly understood how cortical networks are transiently configured to support fast sensorimotor acts …'

    Which implies that the paper will address the mechanism of transient synchronization for fast sensorimotor actions. This is misleading considering the descriptive nature of the results to follow. Being that the results are novel and describe an unresolved phenomenon, an alternative framing emphasizing this would be both compelling and faithful to the work.

2. Near zero connectivity estimates following symmetric orthogonalization are used to rule out signal leakage as an explanation for the wPLI effects (See Supplementary figure S4). However, the aggressive orthogonalization removes both conduction artefacts and genuine coupling from the signal. This makes the result uninformative regarding the extent of conduction artefacts in the non-orthogonalized signal. Using a more sparing orthogonalization scheme would be appropriate.
3. Please report exact number of males and females rather than percentages.

Ethics Related

Name of local ethics committee needs to be mentioned. Does the work really abide Declaration of Helsinki which requires dissemination of de-identified data in public data bases?

Data related

Raw data or preprocessed EEG data couldn't be located. Please identify them with appropriate names. Presentation of only meta-data like phase-lagged index matrices are not acceptable in isolation.
