# Supplementary material for: Frequency-specific cortical subnetworks support fast human swallowing
Source: Commun Biol. 2026 Aug 7;9:1059. doi: 10.1038/s42003-026-10751-6 (PMC13451196; doi:10.1038/s42003-026-10751-6)
Supplement: Supplementary file 3 [file 42003_2026_10751_MOESM2_ESM.pdf]

Supplementary Information

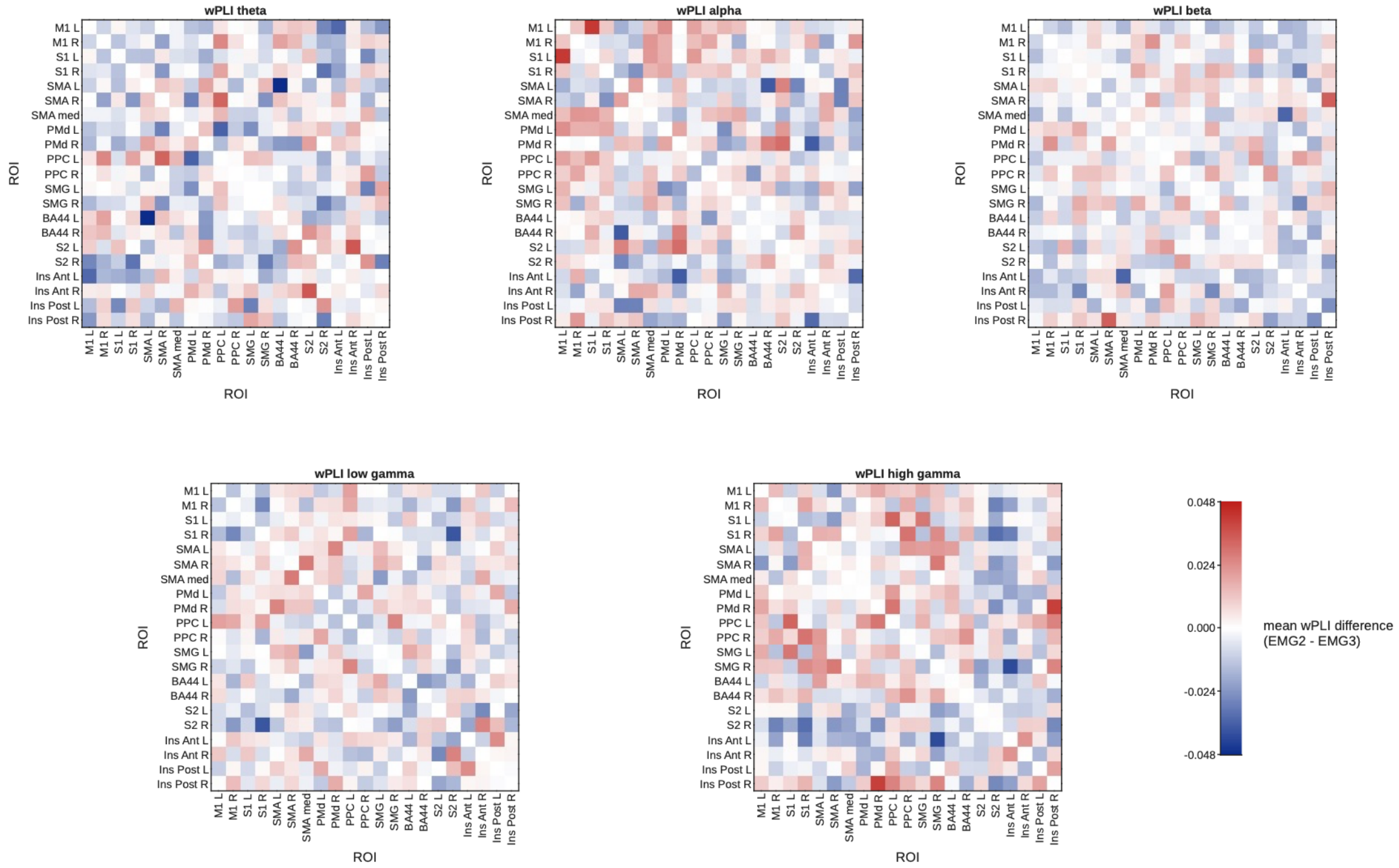

Supplementary Figure 1 Exploratory ROI×ROI wPLI connectivity matrices across frequency bands. Group-level ROI×ROI matrices illustrating mean swallowing-related wPLI connectivity differences (EMG2 – EMG3) across theta, alpha, beta, low-gamma, and high-gamma bands. Warmer colours indicate relatively stronger phase-lagged coupling during swallowing, whereas cooler colours indicate lower coupling relative to the control condition. These matrices provide a descriptive overview of the distributed connectivity structure underlying the exploratory whole-network visualizations shown in Figure 3. Sample size: n = 74 participants.

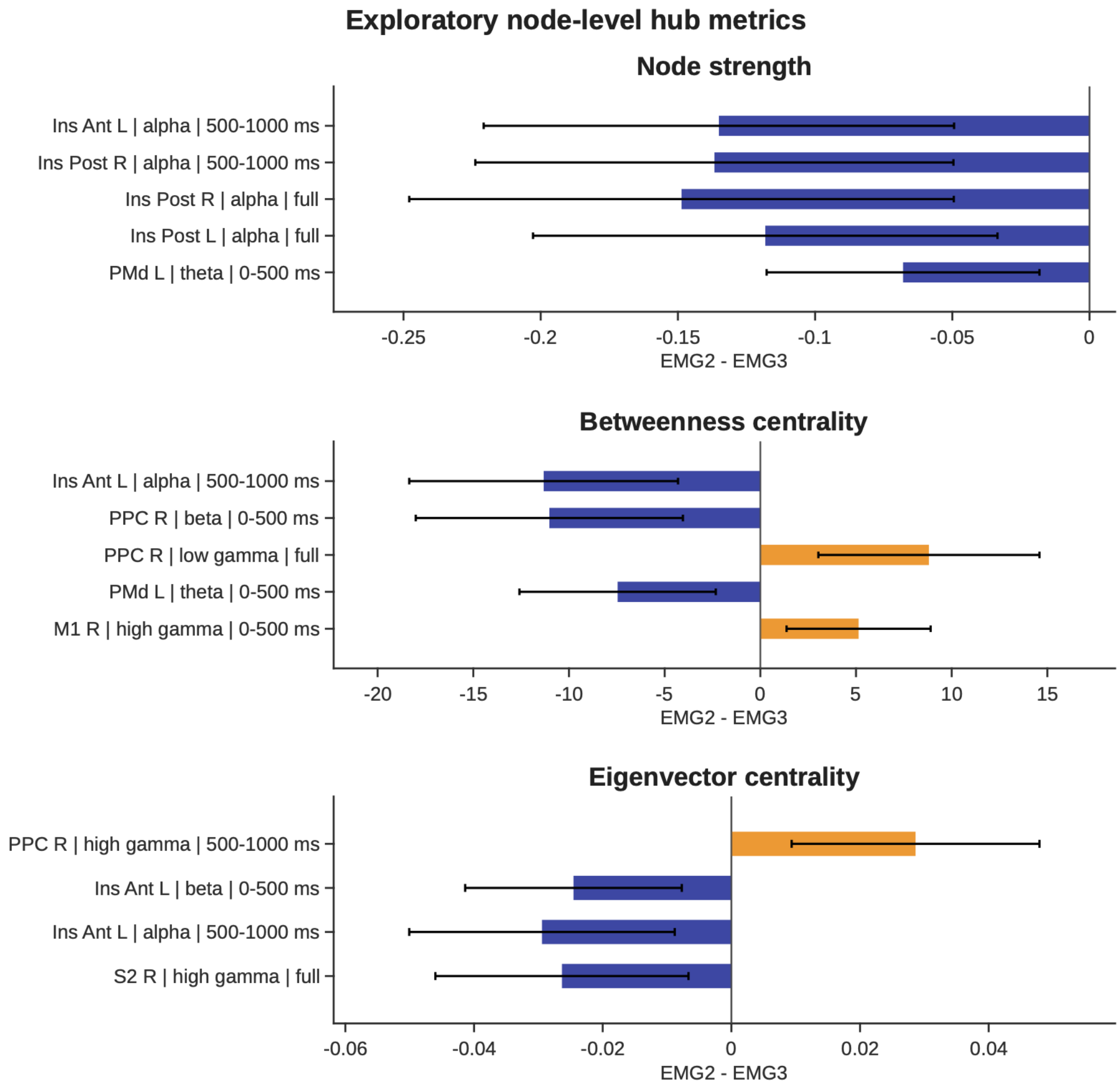

Displayed are the strongest uncorrected node-level effects per metric. No hub/node effect survived FDR correction.

Supplementary Figure 2 Exploratory node-level hub metrics. Exploratory node-level differences in graph-theoretical hub metrics between swallowing (EMG2) and rest (EMG3), including node strength, betweenness centrality, and eigenvector centrality. Displayed effects represent the strongest uncorrected node-level contrasts across frequency bands and time windows. Negative values indicate lower metric values during swallowing relative to rest. Error bars represent 95% confidence intervals. No node-level effect survived false discovery rate correction. Sample size: n = 74 participants.

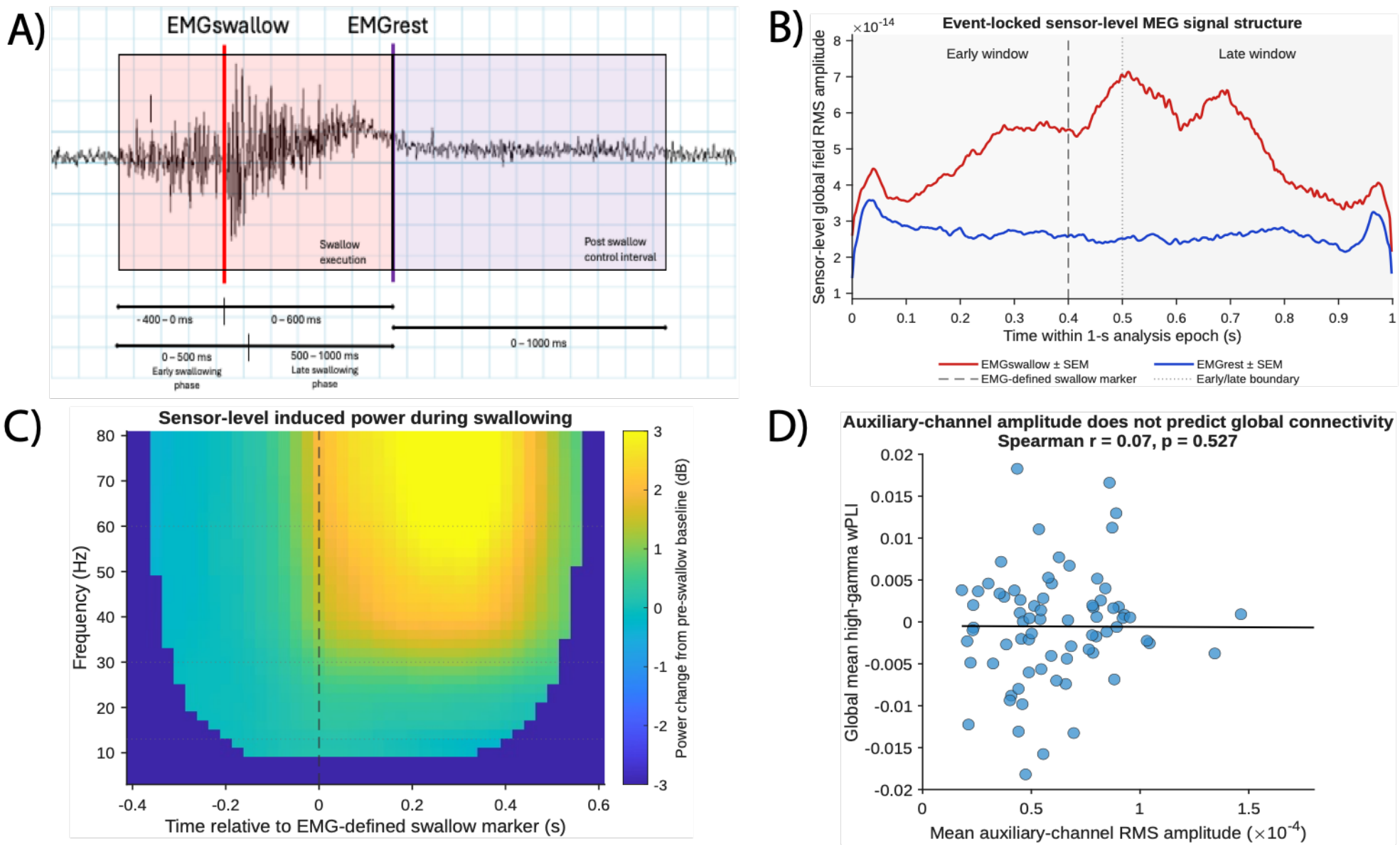

Supplementary Figure 3 Quality-control analyses and preprocessing framework. (A) Illustration of the event-locked swallowing and control epochs derived from submental EMG recordings. (B) Sensor-level MEG signal structure across the 1-s analysis epoch. (C) Time–frequency representation of induced swallowing-related sensor-level power changes. (D) Correlation analysis between auxiliary-channel RMS amplitude and global high-gamma wPLI connectivity estimates demonstrating the absence of a significant association between potential muscle-related signal amplitude and connectivity measures. Together, these analyses support the robustness of the preprocessing strategy and argue against nonspecific muscle-related contamination as the primary explanation for the observed connectivity findings. Sample size: n = 74 participants.

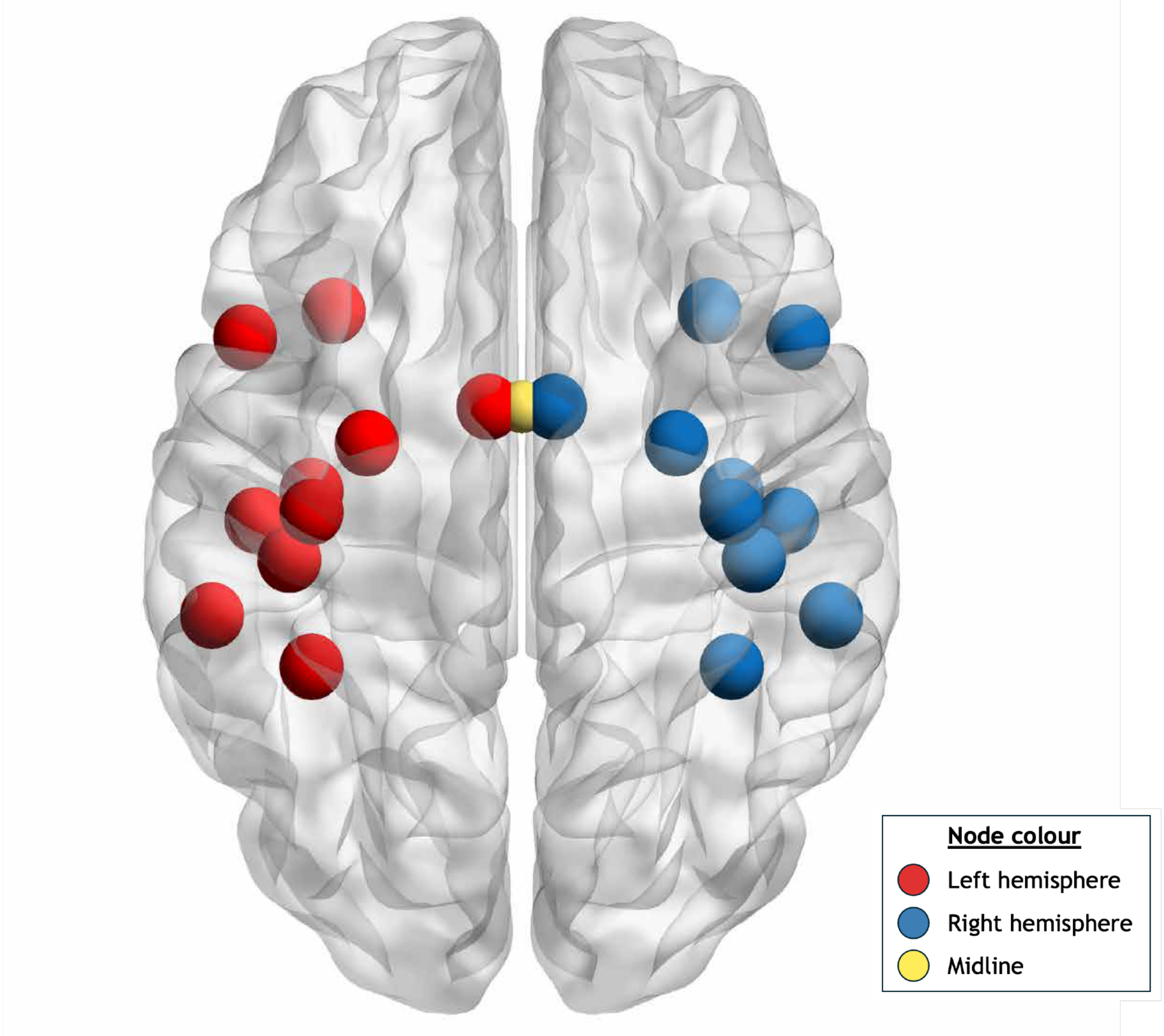

Supplementary Figure 4 Cortical ROI configuration used for source-level connectivity analysis. Spatial distribution of the 21 predefined cortical regions of interest (ROIs) projected onto a standardized inflated cortical surface. ROIs comprised bilateral primary motor cortex (M1), primary and secondary somatosensory cortex (S1, S2), dorsal premotor cortex (PMd), supplementary motor area (SMA), inferior frontal gyrus (BA44), posterior parietal cortex (PPC), supramarginal gyrus (SMG), and anterior and posterior insula. Spherical ROIs (10-mm radius) were centred on predefined MNI coordinates and sampled on the warped template grid used for LCMV beamforming. Node colours indicate anatomical location: red = left hemisphere, blue = right hemisphere, and yellow = midline (SMAmed).

Supplementary Table 1

Nr.	ROI Name	Full Name	Hemisphere	MNI coordinates (x, y, z)
**1**	M1_L	Primary Motor Cortex	Left	–38, –20, 60
**2**	M1_R	Primary Motor Cortex	Right	38, –20, 60
**3**	S1_L	Primary Somatosensory Cortex	Left	–40, –30, 60
**4**	S1_R	Primary Somatosensory Cortex	Right	40, –30, 60
**5**	SMA_L	Supplementary Motor Area	Left	–6, 0, 66
**6**	SMA_R	Supplementary Motor Area	Right	6, 0, 66
**7**	SMA_med	Medial Supplementary Motor Area	Medial	0, 10, 48
**8**	PMd_L	Dorsal Premotor Cortex (BA6)	Left	–41, – 13, 36
**9**	PMd_R	Dorsal Premotor Cortex (BA6)	Right	41, – 13, 36
**10**	PPC_L	Posterior Parietal Cortex (IPL)	Left	–26, –60, 50
**11**	PPC_R	Posterior Parietal Cortex (IPL)	Right	26, –60, 50
**12**	SMG_L	Supramarginal Gyrus	Left	–38, –34, 50
**13**	SMG_R	Supramarginal Gyrus	Right	38, –34, 50
**14**	BA44_L	Inferior Frontal Gyrus, Pars opercularis (BA44)	Left	–44, 26, 30
**15**	BA44_R	Inferior Frontal Gyrus, Pars opercularis (BA44)	Right	44, 26, 30
**16**	S2_L	Secondary Somatosensory Cortex	Left	–56, –42, 22
**17**	S2_R	Secondary Somatosensory Cortex	Right	56, –42, 22
**18**	Ins_Ant_L	Anterior Insula	Left	–34, 18, 2
**19**	Ins_Ant_R	Anterior Insula	Right	34, 18, 2
**20**	Ins_Post_L	Posterior Insula	Left	–36, –18, 14
**21**	Ins_Post_R	Posterior Insula	Right	36, –18, 14
