# Supplementary material for: Frequency-specific cortical subnetworks support fast human swallowing
Source: Commun Biol. 2026 Aug 7;9:1059. doi: 10.1038/s42003-026-10751-6 (PMC13451196; doi:10.1038/s42003-026-10751-6)
Supplement: Supplementary file 4 [file 42003_2026_10751_MOESM3_ESM.pdf]

Descriptions of Additional Supplementary Files

Supplementary Data 1 - Cluster-based permutation statistics underlying the significant subnetworks shown in **Figure 2**.

Supplementary Data 2 - Complete seed-to-ROI statistical results underlying the exploratory connectivity analyses shown in **Figures 3 and 4**.

Supplementary Data 3 - Exploratory FDR-corrected edgewise statistical results supporting the exploratory connectivity analyses.

Supplementary Data 4 - Laterality index analyses underlying **Figure 2F**.

Supplementary Data 5 - Graph-theoretical metrics and statistical results underlying **Figure 5**.

Supplementary Data 6 - Global surrogate robustness analyses supporting the robustness of the whole-network connectivity analyses.

Supplementary Data 7 - ROI definitions and MNI coordinates used for source-level connectivity analyses.
